# Supplementary material for: Perinatal colonization with extended-spectrum beta-lactamase-producing and carbapenem-resistant gram-negative bacteria among home births in Bangladesh
Source: PLoS One. 2025 Sep 19;20(9):e0325404. doi: 10.1371/journal.pone.0325404 (PMC12448980; doi:10.1371/journal.pone.0325404)
Supplement: S2 File — (ZIP) [file pone.0325404.s002.zip › S2_Table 1.docx]

**S2 Table 1:** Complete list of survey questions and responses of mothers undergoing home-based deliveries, Baliakandi, Bangladesh, 2022 (N=50)

| Question | Yes, N (%) | No, N (%) |
| --- | --- | --- |
| What is the mother's age? |  |  |
| At or below median (23) | 27 (54%) | 23 (46%) |
| Above median | 23 (46%) | 27 (54%) |
| How many newborns did the mother give birth to? |  |  |
| 1 newborn | 50 (100%) | 0 (0%) |
| What is the sex of the newborn(s)? |  |  |
| Male | 24 (48%) | 26 (52%) |
| Female | 26 (52%) | 24 (48%) |
| What is the mother's highest level of education? |  |  |
| Primary school | 14 (28%) | 36 (72%) |
| Secondary school | 20 (40%) | 30 (60%) |
| College | 6 (12%) | 44 (88%) |
| Bachelor's degree | 1 (2%) | 49 (98%) |
| Able to sign name only | 7 (14%) | 43 (86%) |
| Unable to sign name | 2 (4%) | 48 (96%) |
| What is the mother's occupation? |  |  |
| Family caregiver | 48 (96%) | 2 (4%) |
| Salaried Job / Government | 1 (2%) | 49 (98%) |
| Tailor | 1 (2%) | 49 (98%) |
| What is the household family income? |  |  |
| At or below median (10,000) | 28 (56%) | 22 (44%) |
| Above median | 22 (44%) | 28 (56%) |
| What was the mode of delivery? |  |  |
| Spontaneous, Vaginal | 50 (100%) | 0 (0%) |
| What was the timing of the delivery (Preterm, full term, or postterm)? |  |  |
| Preterm (<37 weeks) | 3 (6%) | 47 (94%) |
| Full Term (37 weeks) | 46 (92%) | 4 (8%) |
| Postterm (42 weeks) | 1 (2%) | 49 (98%) |
| How many prenatal visits did the mother take? |  |  |
| None | 12 (24%) | 38 (76%) |
| 1-3 visits | 30 (60%) | 20 (40%) |
| 4 or more | 8 (16%) | 42 (84%) |
| Did the mother experience no complications during pregnancy? | 34 (68%) | 16 (32%) |
| Did the mother experience high blood pressure during pregnancy? | 1 (2%) | 49 (98%) |
| Did the mother experience gestational diabetes during pregnancy? | 0 (0%) | 50 (100%) |
| Did the mother experience being underweight during pregnancy? | 1 (2%) | 49 (98%) |
| Did the mother experience bleeding during pregnancy? | 0 (0%) | 50 (100%) |
| Did the mother experience anemia during pregnancy? | 12 (24%) | 38 (76%) |
| Did the mother experience preterm labor during pregnancy? | 0 (0%) | 50 (100%) |
| Did the mother contract HIV during pregnancy? | 0 (0%) | 50 (100%) |
| Did the mother experience urinary infection during pregnancy? | 6 (12%) | 44 (88%) |
| Did the mother experience other infections during pregnancy? | 0 (0%) | 50 (100%) |
| Did the mother experience other complications during pregnancy? | 0 (0%) | 50 (100%) |
| Did the mother use any medications during pregnancy? | 24 (48%) | 26 (52%) |
| Did the mother use any antibiotics during pregnancy? | 4 (8%) | 20 (40%) |
| Was the mother hospitalized during pregnancy? | 1 (2%) | 49 (98%) |
| If yes, for what reason was the mother hospitalized? |  |  |
| Anemia | 1 (2%) | 49 (98%) |
| Did the mother have any prior pregnancies? |  |  |
| None | 18 (36%) | 32 (64%) |
| One | 12 (24%) | 38 (76%) |
| Two | 11 (22%) | 39 (78%) |
| Three or more | 9 (18%) | 41 (82%) |
| How many prior deliveries did the mother have? |  |  |
| None | 18 (36%) | 32 (64%) |
| One | 13 (26%) | 37 (74%) |
| Two | 13 (26%) | 37 (74%) |
| Three or more | 6 (12%) | 44 (88%) |
| How many prior miscarriages did the mother have? |  |  |
| None | 25 (50%) | 25 (50%) |
| One | 6 (12%) | 44 (88%) |
| Two | 1 (2%) | 49 (98%) |
| How many children did the mother have prior to delivery? |  |  |
| Two | 16 (32%) | 34 (68%) |
| Three or more | 16 (32%) | 34 (68%) |
| What is the mother's household size? |  |  |
| 5 or below | 22 (44%) | 28 (56%) |
| 6 or more | 28 (56%) | 22 (44%) |
| Is the mother's primary water source a lake or river? | 0 (0%) | 50 (100%) |
| Is the mother's primary water source an unprotected well? | 0 (0%) | 50 (100%) |
| Is the mother's primary water source a protected well? | 0 (0%) | 50 (100%) |
| Is the mother's primary water source rainwater? | 0 (0%) | 50 (100%) |
| Is the mother's primary water source a tanker truck? | 0 (0%) | 50 (100%) |
| Is the mother's primary water source bottled water? | 0 (0%) | 50 (100%) |
| Is the mother's primary water source shared tap water? | 0 (0%) | 50 (100%) |
| Is the mother's primary water source a tube well? | 49 (98%) | 1 (2%) |
| Is the mother's primary water source a piped household? | 1 (2%) | 49 (98%) |
| Did the mother use no water treatment? | 49 (98%) | 1 (2%) |
| Did the mother use straining through a cloth as water treatment? | 2 (4%) | 48 (96%) |
| Did the mother use boiling as water treatment? | 0 (0%) | 50 (100%) |
| Did the mother use a water filter as water treatment? | 0 (0%) | 50 (100%) |
| Did the mother use sunlight exposure as water treatment? | 0 (0%) | 50 (100%) |
| Did the mother use UV irradiation as water treatment? | 0 (0%) | 50 (100%) |
| Did the mother use chlorine bleach or iodine as water treatment? | 0 (0%) | 50 (100%) |
| Did the mother use any other form of water treatment? | 0 (0%) | 50 (100%) |
| How frequent is this water treatment used? |  |  |
| Often (50-75%) | 1 (2%) | 49 (98%) |
| What is the mother's method of water storage? |  |  |
| Open Container | 3 (6%) | 47 (94%) |
| Narrow Opening Closed Container | 23 (46%) | 27 (54%) |
| Wide Opening Closed Container | 23 (46%) | 27 (54%) |
| What type of toilet facilities are used by the mother? |  |  |
| Shared pit latrine | 24 (48%) | 26 (52%) |
| Private pit latrine | 26 (52%) | 24 (48%) |
| Does the mother use handwashing facilities? | 50 (100%) | 0 (0%) |
| Did the mother have no indoor contact with animals? | 15 (30%) | 35 (70%) |
| Did the mother have indoor contact with chickens? | 15 (30%) | 35 (70%) |
| Did the mother have indoor contact with ducks? | 6 (12%) | 44 (88%) |
| Did the mother have indoor contact with goats? | 3 (6%) | 47 (94%) |
| Did the mother have indoor contact with cows? | 1 (2%) | 49 (98%) |
| Did the mother have indoor contact with sheep? | 0 (0%) | 50 (100%) |
| Did the mother have indoor contact with pigs? | 0 (0%) | 50 (100%) |
| Did the mother have indoor contact with cats? | 32 (64%) | 18 (36%) |
| Did the mother have indoor contact with dogs? | 5 (10%) | 45 (90%) |
| Did the mother have indoor contact with other animals? | 0 (0%) | 50 (100%) |
| Did the mother have no outdoor contact with animals? | 0 (0%) | 50 (100%) |
| Did the mother have outdoor contact with chickens? | 48 (96%) | 2 (4%) |
| Did the mother have outdoor contact with ducks? | 45 (90%) | 5 (10%) |
| Did the mother have outdoor contact with goats? | 33 (66%) | 17 (34%) |
| Did the mother have outdoor contact with cows? | 34 (68%) | 16 (32%) |
| Did the mother have outdoor contact with sheep? | 2 (4%) | 48 (96%) |
| Did the mother have outdoor contact with pigs? | 1 (2%) | 49 (98%) |
| Did the mother have outdoor contact with cats? | 50 (100%) | 0 (0%) |
| Did the mother have outdoor contact with dogs? | 49 (98%) | 1 (2%) |
| Did the mother have outdoor contact with other animals? | 0 (0%) | 50 (100%) |
| Did the mother consume no animal products? | 1 (2%) | 49 (98%) |
| Did the mother consume animal products containing eggs? | 47 (94%) | 3 (6%) |
| Did the mother consume animal products containing dairy milk? | 47 (94%) | 3 (6%) |
| Did the mother consume animal products containing chicken? | 45 (90%) | 5 (10%) |
| Did the mother consume animal products containing beef? | 34 (68%) | 16 (32%) |
| Did the mother consume animal products containing goat? | 8 (16%) | 42 (84%) |
| Did the mother consume animal products containing sheep? | 0 (0%) | 50 (100%) |
| Did the mother consume animal products containing pork? | 0 (0%) | 50 (100%) |
| Did the mother consume animal products containing fish? | 39 (78%) | 11 (22%) |
| Did the mother consume animal products containing shellfish? | 0 (0%) | 50 (100%) |
| Did the mother consume animal products containing duck? | 0 (0%) | 0 (0%) |
| Did the mother consume raw produce? | 49 (98%) | 1 (2%) |
| bv_esbl = vaginal colonization with ESBL organism post-delivery | 26 (52%) | 24 (48%) |
| br_esbl = rectal colonization with ESBL organism post-delivery | 47 (94%) | 3 (6%) |
| c_esbl = newborn colonization with ESBL organism post-delivery | 36 (72%) | 14 (28%) |
| bv_cre = vaginal colonization with carbapenem-resistant organism post-delivery | 27 (54%) | 23 (46%) |
| br_cre = rectal colonization with carbapenem-resistant organism post-delivery | 36 (72%) | 14 (28%) |
| c_cre = newborn colonization with carbapenem-resistant organism post-delivery | 27 (54%) | 23 (46%) |
