# Supplementary material for: Perinatal colonization with extended-spectrum beta-lactamase-producing and carbapenem-resistant gram-negative bacteria among home births in Bangladesh
Source: PLoS One. 2025 Sep 19;20(9):e0325404. doi: 10.1371/journal.pone.0325404 (PMC12448980; doi:10.1371/journal.pone.0325404)
Supplement: S2 File — (ZIP) [file pone.0325404.s002.zip › S2_Table 2.docx]

**S2 Table 2:** Birth attendant practices among home-based delivery, Baliakandi, Bangladesh, 2022 (N=9)

| **Variables** | **n** | **% or range** |
| --- | --- | --- |
| Median age | 52 | 30-60 |
| Highest education completed |  |  |
| No formal education | 1 | 11.1 |
| Able to sign name | 1 | 11.1 |
| Secondary school | 5 | 55.6 |
| College (equivalent to the final 2 years of high school) | 2 | 22.2 |
| Type of training received |  |  |
| Skilled Birth Attendant Training (BRAC or government training) | 4 | 44.4 |
| Informal/ short training | 3 | 33.3 |
| No training | 2 | 22.2 |
| Working with a village doctor/physician |  |  |
| Yes | 3 | 33.3 |
| No | 6 | 66.6 |
| Receive payment for working as a birth attendant | 9 | 100 |
| Average number of years working as a birth attendant | 16 | 6-35 |
| Average number of deliveries per month | 10 | 0-22 |
| Perform deliveries in a facility | 2 | 22.2 |
| Clinic | 1 | 11.1 |
| Birthing center | 1 | 11.1 |
| Conducting prenatal visit |  |  |
| Yes | 9 | 100 |
| No | 0 | 0 |
| How long spent at a delivery |  |  |
| The entire duration of labor | 6 | 66.6 |
| Last stages only | 3 | 33.3 |
| Average number of prenatal visits conducted for a pregnant woman | 3.3 | 1-6 |
| Equipment/supplies usually taken along for delivery |  |  |
| Gloves | 8 | 88.8 |
| Soap | 4 | 44.4 |
| Scissors | 4 | 44.4 |
| Alcohol-based hand rub | 4 | 44.4 |
| Oral medications | 4 | 44.4 |
| Plastic sheets | 3 | 33.3 |
| Sutures | 3 | 33.3 |
| Injectable medications | 3 | 33.3 |
| Cloths, towels, or drapes | 3 | 33.3 |
| Skin sterilizing agent | 2 | 22.2 |
| Gown | 2 | 22.2 |
| Reused equipment/supplies |  |  |
| Scissors | 4 | 44.4 |
| Plastic sheets | 3 | 33.3 |
| Blood pressure cuff | 3 | 33.3 |
| Thermometer | 2 | 22.2 |
| Cloth sheets, towels, or drapes | 1 | 11.1 |
| Gown | 1 | 11.1 |
| Forceps | 1 | 11.1 |
| Stethoscope | 1 | 11.1 |
| Cleaning modalities for reuse of scissors (multiple responses) |  |  |
| Cleaned with water | 2 | 22.2 |
| Boiled | 1 | 11.1 |
| Cleaned with soap and water | 1 | 11.1 |
| Not cleaned | 1 | 11.1 |
| Cleaning modalities for reuse of plastic sheets (multiple responses) |  |  |
| Cleaned with water | 2 | 22.2 |
| Cleaned with water and soap | 1 | 11.1 |
| Boiled | 1 | 11.1 |
| Cleaning modalities for reuse of blood pressure cuffs (multiple responses) |  |  |
| Not cleaned | 2 | 22.2 |
| Cleaning modalities for reuse of thermometer (multiple responses) |  |  |
| Cleaned with water | 1 | 11.1 |
| Not cleaned | 1 | 11.1 |
| Cleaning modalities for reuse of cloths, towels, or drapes (multiple responses) |  |  |
| Cleaned with water and soap | 1 | 11.1 |
| Not cleaned | 1 | 11.1 |
| Cleaning modalities for reuse of gowns (multiple responses) |  |  |
| Cleaned with water | 1 | 11.1 |
| Cleaned with water and soap | 1 | 11.1 |
| Cleaning modalities for reuse of forceps (multiple responses) |  |  |
| Cleaned with water and soap | 1 | 11.1 |
| Cleaning modalities for reuse of stethoscope (multiple responses) |  |  |
| Not cleaned | 2 | 22.2 |
| Household places where delivery is usually conducted |  |  |
| In living room or kitchen area | 7 | 77.7 |
| In a separate room which is mostly unused | 1 | 11.1 |
| In bedroom | 1 | 11.1 |
| Family members present during delivery |  |  |
| Mother-in-law | 9 | 100 |
| Aunt | 8 | 88.9 |
| Mother | 6 | 66.6 |
| Sister-in-law | 4 | 44.4 |
| Sister | 3 | 33.3 |
| Average number of people present during labor/delivery | 3.2 | 2-5 |
| Preparation taken before the delivery procedure |  |  |
| Performing hand hygiene with soap and water | 7 | 77.7 |
| Donning gloves | 7 | 77.7 |
| Obtaining water from a local source | 6 | 66.6 |
| Disinfecting delivery area | 4 | 44.4 |
| Laying out clothes or blankets | 4 | 44.4 |
| Laying out plastic sheets | 3 | 33.3 |
| Performing hand hygiene with alcohol-based hand rub | 1 | 11.1 |
| Use of treated water for delivery |  |  |
| Yes | 8 | 88.8 |
| No | 1 | 11.1 |
| Process of water treatment |  |  |
| Boiling | 8 | 88.8 |
| Straining through cloth | 2 | 22.2 |
| Hand hygiene is performed |  |  |
| Before cervical checks | 8 | 88.8 |
| Before any procedure | 6 | 66.6 |
| Before wearing gloves | 6 | 66.6 |
| Before touching the mother | 3 | 33.3 |
| Before catching the baby | 3 | 33.3 |
| After removing gloves | 2 | 22.2 |
| After touching the mother | 2 | 22.2 |
| After body fluid exposure | 2 | 22.2 |
| After cervical checks | 1 | 11.1 |
| After catching the baby | 1 | 11.1 |
| Gloves are used |  |  |
| Throughout the entire procedure | 5 | 55.5 |
| For cervical checks | 5 | 55.5 |
| For delivering the baby | 5 | 55.5 |
| For any procedure | 2 | 22.2 |
| Do not use gloves | 1 | 11.1 |
| Cervical checks |  |  |
| Yes | 8 | 88.8 |
| No | 1 | 11.1 |
| Frequency of cervical checks |  |  |
| 6 or more times | 3 | 33.3 |
| 4-5 times | 2 | 22.2 |
| 3-2 times | 2 | 22.2 |
| Once | 1 | 11.1 |
| Membrane stripping/sweeping |  |  |
| Yes | 3 | 33.3 |
| No | 6 | 66.66 |
| Mechanical cervical ripening |  |  |
| Yes | 0 | 0 |
| No | 9 | 100 |
| Artificial rupture of membranes |  |  |
| Yes | 1 | 11.1 |
| No | 8 | 88.9 |
| Perform episiotomies |  |  |
| Never | 7 | 77.8 |
| Rarely | 2 | 22.2 |
| Administration of medication to mothers |  |  |
| Yes | 7 | 77.7 |
| No | 2 | 22.2 |
| Medications administered to mothers |  |  |
| Painkiller (oral) | 5 | 55.5 |
| Oxytocin (IV) | 4 | 44.4 |
| Antibiotics (oral) | 3 | 33.3 |
| Oxytocin (IM) | 3 | 33.3 |
| Misoprostol (oral) | 1 | 11.1 |
| Others (anti-ulcerate, suppository for pain) | 3 | 33.3 |
| Reason for antibiotic administration to mothers |  |  |
| Prophylactic | 2 | 22.2 |
| Fever | 1 | 11.1 |
| Administration of medication to babies |  |  |
| Yes | 3 | 33.3 |
| No | 6 | 66.6 |
| Type of medication administered to babies |  |  |
| Antibiotics (oral) | 2 | 22.2 |
| Antibiotics (injectable) | 2 | 22.2 |
| Vitamin K | 2 | 22.2 |
| Others (analgesics) | 1 | 11.1 |
| Reason for antibiotic administration to babies |  |  |
| Fever | 2 | 22.2 |
| Other (cold) | 1 | 11.1 |
| Means of cutting umbilical cord |  |  |
| By sterile knife/scalpel | 3 | 33.3 |
| By sterile scissors | 2 | 22.2 |
| By non-sterile scissors | 1 | 11.1 |
| With a new blade | 2 | 22.2 |
| Immediate newborn management |  |  |
| Place baby on mother's breast | 8 | 88.8 |
| Wipe/dry baby | 7 | 77.7 |
| Clean baby's mouth | 7 | 77.7 |
| Stimulate baby by slapping/shaking | 4 | 44.4 |
| Skin-to-skin contact | 1 | 11.1 |
| Cord management |  |  |
| Application of chlorhexidine | 7 | 77.7 |
| Dry cord care | 4 | 44.4 |
| Covering with clean cloths | 1 | 11.1 |
| Cleaning the baby's mouth after delivery |  |  |
| With a cloth/gauze | 8 | 88.8 |
| Finger sweep without gloves | 2 | 22.2 |
| Finger sweep with gloves | 1 | 11.1 |
| Penguin sucker | 1 | 11.1 |
