# Supplementary material for: Perinatal colonization with extended-spectrum beta-lactamase-producing and carbapenem-resistant gram-negative bacteria among home births in Bangladesh
Source: PLoS One. 2025 Sep 19;20(9):e0325404. doi: 10.1371/journal.pone.0325404 (PMC12448980; doi:10.1371/journal.pone.0325404)
Supplement: S2 File — (ZIP) [file pone.0325404.s002.zip › S2_Figure 1.docx]

**S2 Figure 1:** Extended-spectrum beta-lactamase-producing bacterial colonization prevalence by presumptive bacterial identification among mothers and newborns following home-based delivery, Baliakandi, Bangladesh, 2022 (N=50)


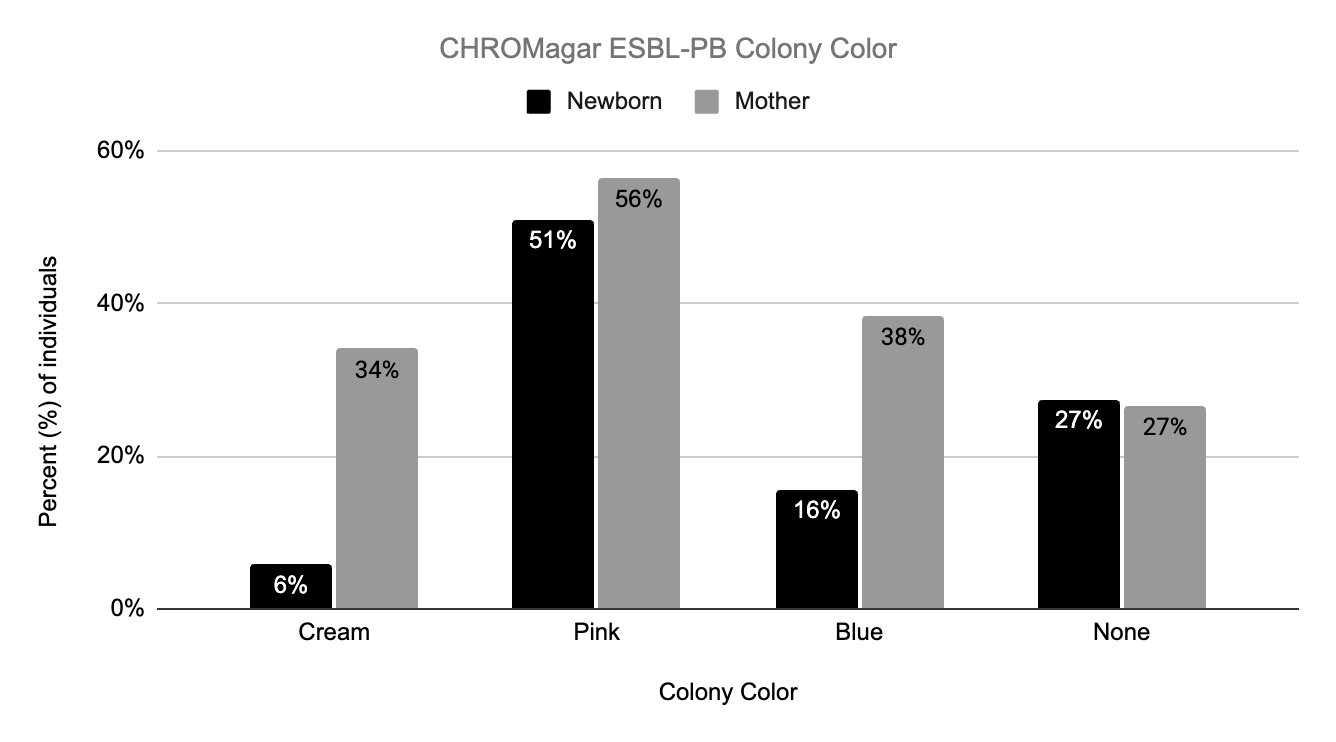


Presumptive colony ID: cream = *Pseudomonas* or *Acinetobacter* spp.; pink = *E. coli*; blue = *Klebsiella, Enterobacter,* or *Citrobacter* spp.; none = no growth
